# *catena*-Poly[bis­(propane-1,3-diaminium) [[aqua­(sulfato-κ*O*)bis­(sulfato-κ^2^ *O*,*O*′)cerate(IV)]-μ-sulfato-κ^3^ *O*,*O*′:*O*′′] dihydrate]

**DOI:** 10.1107/S1600536811008324

**Published:** 2011-03-09

**Authors:** Ali Farooq Meer, Saeed Ahmad, Shahzad Sharif, Islam Ullah Khan, Seik Weng Ng

**Affiliations:** a Department of Chemistry, University of Engineering and Technology, Lahore 54890, Pakistan; b Materials Chemistry Laboratory, Department of Chemistry, Government College University, 54000 Lahore, Pakistan; c Department of Chemistry, University of Malaya, 50603 Kuala Lumpur, Malaysia

## Abstract

The Ce^IV^ atom in the title salt, {(H_3_NCH_2_CH_2_CH_2_NH_3_)_2_[Ce(SO_4_)_4_(H_2_O)]·2H_2_O}_*n*_, exists in a monocapped square-anti­prismatic coordination geometry. The water-coordinated metal atom is bonded to four sulfate ions; one of them is monodentate and two function in a chelating mode. The fourth is also chelating but it uses one of the other two O atoms to bind to an adjacent metal atom, generating a polyanionic chain. The cations are linked to the polyanionic chain as well as to the uncoordinated water mol­ecules, resulting in an O—H⋯O and N—H⋯O hydrogen-bonded three-dimensional network.

## Related literature

For (C_2_H_10_N_2_)_5_[Ce_2_(SO_4_)_9_]^.^3H_2_O, see: Jabeen *et al.* (2010 ▶).
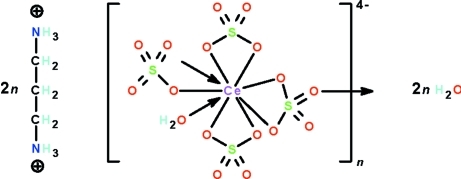


## Experimental

### 

#### Crystal data

(C_3_H_12_N_2_)_2_[Ce(SO_4_)_4_(H_2_O)]·2H_2_O
*M* *_r_* = 730.70
Monoclinic, 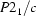

*a* = 8.9459 (1) Å
*b* = 20.4497 (3) Å
*c* = 12.8688 (2) Å
β = 99.535 (1)°
*V* = 2321.71 (6) Å^3^
*Z* = 4
Mo *K*α radiation
μ = 2.41 mm^−1^
*T* = 295 K
0.30 × 0.15 × 0.10 mm

#### Data collection

Bruker Kappa APEXII diffractometer
Absorption correction: multi-scan (*SADABS*; Sheldrick, 1996 ▶) *T*_min_ = 0.531, *T*_max_ = 0.794
21238 measured reflections
5296 independent reflections
4663 reflections with *I* > 2σ(*I*)
*R*_int_ = 0.024

#### Refinement

*R*[*F* ^2^ > 2σ(*F* ^2^)] = 0.028
*wR*(*F* ^2^) = 0.071
*S* = 1.14
5296 reflections
307 parameters
H-atom parameters constrained
Δρ_max_ = 1.24 e Å^−3^
Δρ_min_ = −1.41 e Å^−3^

### 

Data collection: *APEX2* (Bruker, 2009 ▶); cell refinement: *SAINT* (Bruker, 2009 ▶); data reduction: *SAINT*; program(s) used to solve structure: *SHELXS97* (Sheldrick, 2008 ▶); program(s) used to refine structure: *SHELXL97* (Sheldrick, 2008 ▶); molecular graphics: *X-SEED* (Barbour, 2001 ▶); software used to prepare material for publication: *publCIF* (Westrip, 2010 ▶).

## Supplementary Material

Crystal structure: contains datablocks global, I. DOI: 10.1107/S1600536811008324/bt5471sup1.cif

Structure factors: contains datablocks I. DOI: 10.1107/S1600536811008324/bt5471Isup2.hkl

Additional supplementary materials:  crystallographic information; 3D view; checkCIF report

## Figures and Tables

**Table 1:** Hydrogen-bond geometry (Å, °)

*D*—H⋯*A*	*D*—H	H⋯*A*	*D*⋯*A*	*D*—H⋯*A*
O1*w*—H1*w*2⋯O12^i^	0.84	2.05	2.854 (4)	162
O2*w*—H2*w*1⋯O11^i^	0.84	2.24	2.935 (5)	140
O2*w*—H2*w*2⋯O15^ii^	0.84	2.08	2.902 (5)	167
O3*w*—H3*w*1⋯O3^i^	0.84	1.90	2.729 (5)	169
O3*w*—H3*w*2⋯O2*w*	0.85	1.98	2.802 (5)	163
N1—H11⋯O9^i^	0.86	2.04	2.881 (4)	167
N1—H12⋯O3*w*	0.86	1.95	2.806 (5)	171
N1—H13⋯O13^iii^	0.86	2.19	3.036 (4)	166
N2—H21⋯O16^iv^	0.86	2.09	2.904 (5)	157
N2—H22⋯O16^ii^	0.86	2.25	2.978 (5)	142
N2—H23⋯O2	0.86	2.49	3.180 (6)	137
N2—H23⋯O5	0.86	2.44	2.989 (4)	122
N3—H31⋯O6^v^	0.86	2.04	2.866 (4)	162
N3—H32⋯O4	0.86	2.37	2.882 (6)	119
N3—H32⋯O2*w*^vi^	0.86	2.37	3.085 (6)	141
N3—H33⋯O1	0.86	2.33	3.004 (5)	135
N3—H33⋯O10	0.86	2.43	3.191 (6)	147
N4—H41⋯O6^vii^	0.86	2.34	2.986 (4)	132
N4—H41⋯O15^vii^	0.86	2.25	2.951 (5)	138
N4—H43⋯O4^viii^	0.86	2.00	2.851 (5)	173

Symmetry codes: (i) 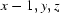; (ii) 
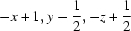
; (iii) 
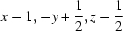
; (iv) 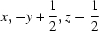; (v) 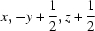; (vi) 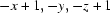; (vii) 
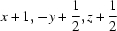
; (viii) 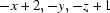.

**Fig. 1.**
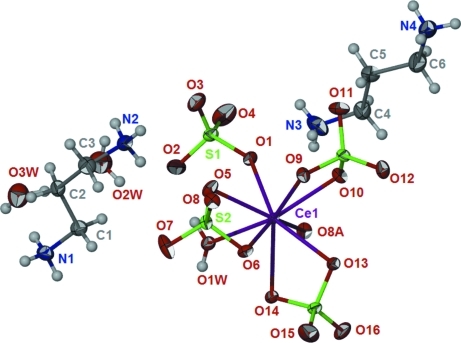
Thermal ellipsoid plot (Barbour, 2001) of a portion of polymeric (H3NCH2CH2CH2NH3) [Ce(H2O)(SO4)4].2H2O at the 50% probability level; hydrogen atoms are drawn as spheres of arbitrary radius.

**Fig. 2.**
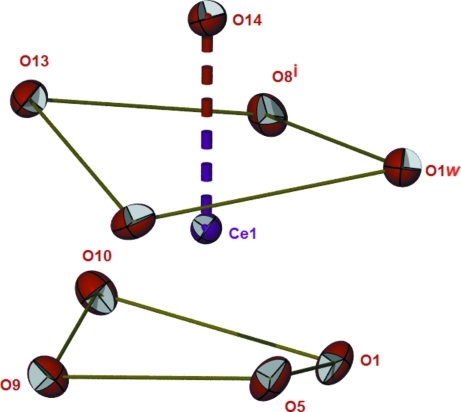
Monocapped square-antiprismatic geometry of Ce(IV).

## supplementary crystallographic information

### Comment

A previous study reports the isolation of pentakis(ethylenediammonium) µ-sulfato-bis[trisulfatocerate(IV) trihydrate, which was synthesized by the reaction of 1,2-diaminoethane with cerium(IV) sulfate (Jabeen *et al.*, 2010). The two independent Ce atoms exist in a nine-coordinate geometry this is best described as a tricapped trigonal prism. Shortening the cationic chain results in the formatoin of a polyanion. The compound obtained with 1,3-diaminopropane in place of 1,2-diamionoethane is 2(H_3_NCH_2_CH_2_CH_2_NH_3_) [Ce(H_2_O)(SO_4_)_4_]^.^2H_2_O (Scheme I, Fig. 1). One of the sulfate ions behaving in a bridging mode to link adjacent cerate ions into a chain. The cations are linked to the polyanionic chain as well as to the lattice water molecules to result in a hydrogen-bonded three-dimensional network. The metal atom shows monocapped square-antiprismatic geometry (Fig. 2).

### Experimental

1,3-Diaminopropane (0.148 g, 2 mmol) was placed in a 1 N sulfuric acid solution (5 ml) of cerium(IV) sulfate tetrahydrate (0.202 g, 0.5 mmol). The yellow solution was filtered and then set aside for the growth of crystals.

### Refinement

Carbon- and nitrogen-bound H-atoms were placed in calculated positions (C–H 0.93 to 0.97 Å; N–H 0.86 Å) and were included in the refinement in the riding model approximation, with *U*(H) set to 1.2–1.5*U*(C,*N*). The H-atoms of the water molecules were placed in chemically sensible positions on the basis of hydrogen bonding interactions (O–H 0.84 Å) and their temperature factors were similar tied. The final difference Fouier map had a peak at 0.85 Å from Ce1 and a hole at 1.18 Å from O10.

The (1 0 0), (1 1 0) and (0 2 0) reflections were omitted as they were affected by the beam stop. The reflections (-1 3 10), (-1 7 8), (0 7 6), (0 1 6), (-1 8 12) and (-1 2 12) were omitted because of bad disagreement between the calculated and observed intensities.

### Crystal data

**Table:**

(C_3_H_12_N_2_)_2_[Ce(SO_4_)_4_(H_2_O)]·2H_2_O	*F*(000) = 1472
*M**_r_* = 730.70	*D*_x_ = 2.090 Mg m^−^^3^
Monoclinic, *P*2_1_/*c*	Mo *K*α radiation, λ = 0.71073 Å
Hall symbol: -P 2ybc	Cell parameters from 9960 reflections
*a* = 8.9459 (1) Å	θ = 2.8–28.3°
*b* = 20.4497 (3) Å	µ = 2.41 mm^−^^1^
*c* = 12.8688 (2) Å	*T* = 295 K
β = 99.535 (1)°	Prism, yellow
*V* = 2321.71 (6) Å^3^	0.30 × 0.15 × 0.10 mm
*Z* = 4	

### Data collection

**Table:**

Bruker Kappa APEXII diffractometer	5296 independent reflections
Radiation source: fine-focus sealed tube	4663 reflections with *I* > 2σ(*I*)
graphite	*R*_int_ = 0.024
φ and ω scans	θ_max_ = 27.5°, θ_min_ = 1.9°
Absorption correction: multi-scan (*SADABS*; Sheldrick, 1996)	*h* = −11→11
*T*_min_ = 0.531, *T*_max_ = 0.794	*k* = −26→20
21238 measured reflections	*l* = −16→16

### Refinement

**Table:**

Refinement on *F*^2^	Primary atom site location: structure-invariant direct methods
Least-squares matrix: full	Secondary atom site location: difference Fourier map
*R*[*F*^2^ > 2σ(*F*^2^)] = 0.028	Hydrogen site location: inferred from neighbouring sites
*wR*(*F*^2^) = 0.071	H-atom parameters constrained
*S* = 1.14	*w* = 1/[σ^2^(*F*_o_^2^) + (0.0167*P*)^2^ + 7.615*P*] where *P* = (*F*_o_^2^ + 2*F*_c_^2^)/3
5296 reflections	(Δ/σ)_max_ = 0.001
307 parameters	Δρ_max_ = 1.24 e Å^−^^3^
0 restraints	Δρ_min_ = −1.40 e Å^−^^3^

### Special details

**Table:**

Geometry. All e.s.d.'s (except the e.s.d. in the dihedral angle between two l.s. planes) are estimated using the full covariance matrix. The cell e.s.d.'s are taken into account individually in the estimation of e.s.d.'s in distances, angles and torsion angles; correlations between e.s.d.'s in cell parameters are only used when they are defined by crystal symmetry. An approximate (isotropic) treatment of cell e.s.d.'s is used for estimating e.s.d.'s involving l.s. planes.

### Fractional atomic coordinates and isotropic or equivalent isotropic displacement parameters (Å^2^)

**Table:**

	*x*	*y*	*z*	*U*_iso_*/*U*_eq_	
Ce1	0.682986 (19)	0.252538 (8)	0.326048 (13)	0.01407 (6)	
S1	0.58776 (10)	0.08286 (4)	0.35192 (8)	0.02668 (19)	
S2	0.57080 (9)	0.24695 (4)	0.08631 (6)	0.01880 (16)	
S3	1.02446 (9)	0.22812 (4)	0.34548 (7)	0.02170 (17)	
S4	0.66130 (10)	0.39960 (4)	0.35617 (7)	0.02449 (18)	
O1	0.6789 (3)	0.14558 (12)	0.3672 (2)	0.0248 (5)	
O2	0.4287 (3)	0.10079 (15)	0.3213 (4)	0.0588 (11)	
O3	0.6376 (4)	0.04469 (15)	0.2697 (3)	0.0429 (7)	
O4	0.6144 (4)	0.04863 (16)	0.4518 (3)	0.0530 (9)	
O5	0.6001 (3)	0.19145 (12)	0.16128 (19)	0.0266 (5)	
O6	0.6232 (3)	0.30497 (11)	0.15445 (18)	0.0227 (5)	
O7	0.4153 (3)	0.25303 (17)	0.0394 (2)	0.0439 (8)	
O8	0.6718 (3)	0.24052 (12)	0.00700 (19)	0.0246 (5)	
O9	0.9026 (3)	0.23795 (12)	0.24892 (19)	0.0236 (5)	
O10	0.9303 (3)	0.23354 (13)	0.4321 (2)	0.0253 (5)	
O11	1.0895 (3)	0.16402 (14)	0.3425 (2)	0.0384 (7)	
O12	1.1351 (3)	0.27980 (14)	0.3520 (2)	0.0323 (6)	
O13	0.8002 (3)	0.35764 (12)	0.3595 (2)	0.0236 (5)	
O14	0.5368 (3)	0.35021 (12)	0.3413 (2)	0.0231 (5)	
O15	0.6465 (3)	0.44422 (15)	0.2677 (3)	0.0486 (8)	
O16	0.6665 (3)	0.43396 (15)	0.4552 (3)	0.0436 (8)	
O1w	0.4185 (3)	0.23056 (12)	0.3117 (2)	0.0245 (5)	
H1w1	0.4039	0.1903	0.3164	0.037*	
H1w2	0.3478	0.2512	0.3315	0.037*	
O2w	0.1571 (4)	0.02367 (19)	0.3392 (3)	0.0621 (10)	
H2w1	0.1834	0.0622	0.3559	0.093*	
H2w2	0.2243	0.0057	0.3107	0.093*	
O3w	−0.1048 (4)	0.02558 (18)	0.1859 (3)	0.0572 (9)	
H3w1	−0.1861	0.0260	0.2112	0.086*	
H3w2	−0.0325	0.0329	0.2360	0.086*	
N1	−0.0516 (4)	0.14453 (17)	0.0895 (3)	0.0332 (7)	
H11	−0.0698	0.1765	0.1290	0.050*	
H12	−0.0780	0.1084	0.1155	0.050*	
H13	−0.1022	0.1498	0.0272	0.050*	
N2	0.4215 (4)	0.07383 (16)	0.0772 (3)	0.0334 (7)	
H21	0.5087	0.0757	0.0575	0.050*	
H22	0.4077	0.0352	0.1003	0.050*	
H23	0.4179	0.1019	0.1264	0.050*	
N3	0.8546 (4)	0.1186 (2)	0.5827 (3)	0.0539 (11)	
H31	0.7883	0.1354	0.6161	0.081*	
H32	0.8346	0.0778	0.5713	0.081*	
H33	0.8522	0.1384	0.5236	0.081*	
N4	1.3989 (4)	0.08791 (18)	0.5938 (3)	0.0445 (9)	
H41	1.4856	0.0974	0.6304	0.067*	
H42	1.3901	0.1068	0.5334	0.067*	
H43	1.3920	0.0463	0.5851	0.067*	
C1	0.1122 (4)	0.14262 (19)	0.0843 (3)	0.0308 (8)	
H1A	0.1699	0.1367	0.1544	0.037*	
H1B	0.1430	0.1836	0.0563	0.037*	
C2	0.1438 (4)	0.0867 (2)	0.0140 (3)	0.0322 (8)	
H2A	0.1307	0.0457	0.0493	0.039*	
H2B	0.0703	0.0879	−0.0505	0.039*	
C3	0.3013 (4)	0.0886 (2)	−0.0138 (3)	0.0348 (9)	
H3A	0.3073	0.0572	−0.0694	0.042*	
H3B	0.3193	0.1317	−0.0407	0.042*	
C4	1.0041 (5)	0.1253 (2)	0.6454 (4)	0.0438 (11)	
H4A	1.0057	0.1038	0.7128	0.053*	
H4B	1.0260	0.1713	0.6587	0.053*	
C5	1.1240 (5)	0.0961 (2)	0.5913 (4)	0.0379 (9)	
H5A	1.1098	0.0491	0.5862	0.045*	
H5B	1.1141	0.1134	0.5204	0.045*	
C6	1.2785 (5)	0.1104 (3)	0.6490 (4)	0.0488 (12)	
H6A	1.2885	0.1573	0.6600	0.059*	
H6B	1.2907	0.0897	0.7177	0.059*	

### Atomic displacement parameters (Å^2^)

**Table:**

	*U*^11^	*U*^22^	*U*^33^	*U*^12^	*U*^13^	*U*^23^
Ce1	0.01607 (9)	0.01389 (10)	0.01262 (9)	−0.00095 (6)	0.00347 (6)	−0.00052 (6)
S1	0.0263 (4)	0.0165 (4)	0.0378 (5)	−0.0020 (3)	0.0068 (4)	0.0034 (4)
S2	0.0183 (4)	0.0248 (4)	0.0135 (3)	−0.0014 (3)	0.0034 (3)	−0.0013 (3)
S3	0.0178 (4)	0.0231 (4)	0.0246 (4)	0.0031 (3)	0.0048 (3)	−0.0003 (3)
S4	0.0238 (4)	0.0151 (4)	0.0346 (5)	0.0002 (3)	0.0049 (3)	−0.0016 (3)
O1	0.0269 (13)	0.0162 (12)	0.0301 (13)	−0.0039 (10)	0.0017 (10)	0.0035 (10)
O2	0.0221 (14)	0.0242 (16)	0.129 (4)	−0.0024 (12)	0.0085 (17)	−0.0031 (18)
O3	0.0468 (17)	0.0337 (16)	0.0485 (19)	−0.0058 (13)	0.0090 (14)	−0.0141 (14)
O4	0.084 (3)	0.0323 (17)	0.0453 (19)	−0.0127 (16)	0.0176 (18)	0.0116 (14)
O5	0.0414 (15)	0.0198 (12)	0.0197 (12)	−0.0089 (11)	0.0079 (10)	−0.0012 (10)
O6	0.0318 (13)	0.0166 (12)	0.0197 (12)	0.0016 (10)	0.0045 (9)	−0.0005 (9)
O7	0.0197 (13)	0.082 (3)	0.0286 (15)	0.0005 (14)	0.0004 (11)	−0.0020 (15)
O8	0.0264 (12)	0.0317 (14)	0.0173 (12)	0.0022 (10)	0.0085 (10)	0.0005 (10)
O9	0.0202 (11)	0.0301 (14)	0.0204 (12)	0.0001 (10)	0.0031 (9)	−0.0020 (10)
O10	0.0210 (12)	0.0319 (14)	0.0230 (13)	0.0031 (10)	0.0039 (9)	0.0037 (10)
O11	0.0404 (16)	0.0291 (15)	0.0460 (18)	0.0150 (12)	0.0087 (13)	0.0000 (13)
O12	0.0199 (12)	0.0370 (16)	0.0395 (16)	−0.0061 (11)	0.0037 (11)	−0.0016 (12)
O13	0.0197 (11)	0.0184 (12)	0.0326 (14)	−0.0008 (9)	0.0041 (10)	−0.0020 (10)
O14	0.0205 (11)	0.0198 (12)	0.0294 (13)	−0.0002 (9)	0.0049 (10)	−0.0038 (10)
O15	0.0407 (17)	0.0327 (17)	0.069 (2)	0.0003 (13)	0.0012 (15)	0.0261 (16)
O16	0.0383 (16)	0.0337 (16)	0.060 (2)	−0.0041 (13)	0.0110 (14)	−0.0263 (15)
O1w	0.0227 (12)	0.0217 (12)	0.0302 (14)	−0.0015 (10)	0.0076 (10)	−0.0023 (10)
O2w	0.049 (2)	0.054 (2)	0.086 (3)	−0.0027 (17)	0.0191 (19)	0.005 (2)
O3w	0.053 (2)	0.064 (2)	0.056 (2)	−0.0085 (18)	0.0129 (17)	−0.0049 (18)
N1	0.0322 (17)	0.0359 (19)	0.0298 (17)	0.0104 (14)	0.0005 (13)	−0.0055 (14)
N2	0.0296 (16)	0.0261 (17)	0.044 (2)	−0.0017 (13)	0.0034 (14)	−0.0044 (15)
N3	0.037 (2)	0.076 (3)	0.047 (2)	0.022 (2)	0.0011 (17)	−0.008 (2)
N4	0.0301 (18)	0.033 (2)	0.073 (3)	−0.0063 (15)	0.0152 (18)	−0.0067 (18)
C1	0.033 (2)	0.029 (2)	0.030 (2)	0.0023 (16)	0.0053 (15)	−0.0011 (16)
C2	0.0249 (18)	0.035 (2)	0.037 (2)	−0.0001 (16)	0.0035 (15)	−0.0076 (17)
C3	0.033 (2)	0.041 (2)	0.031 (2)	0.0027 (17)	0.0079 (16)	−0.0011 (17)
C4	0.035 (2)	0.046 (3)	0.049 (3)	0.0014 (19)	0.0043 (19)	−0.012 (2)
C5	0.037 (2)	0.035 (2)	0.042 (2)	0.0055 (18)	0.0066 (18)	−0.0005 (18)
C6	0.037 (2)	0.049 (3)	0.062 (3)	−0.007 (2)	0.012 (2)	−0.015 (2)

### Geometric parameters (Å, °)

**Table:**

Ce1—O1	2.252 (2)	N1—C1	1.479 (5)
Ce1—O8^i^	2.352 (2)	N1—H11	0.8600
Ce1—O9	2.362 (2)	N1—H12	0.8600
Ce1—O1w	2.385 (2)	N1—H13	0.8600
Ce1—O13	2.399 (2)	N2—C3	1.484 (5)
Ce1—O14	2.413 (2)	N2—H21	0.8600
Ce1—O10	2.431 (2)	N2—H22	0.8600
Ce1—O6	2.433 (2)	N2—H23	0.8600
Ce1—O5	2.467 (2)	N3—C4	1.450 (6)
S1—O3	1.444 (3)	N3—H31	0.8600
S1—O4	1.448 (3)	N3—H32	0.8600
S1—O2	1.459 (3)	N3—H33	0.8600
S1—O1	1.515 (2)	N4—C6	1.460 (6)
S2—O7	1.427 (3)	N4—H41	0.8600
S2—O8	1.477 (2)	N4—H42	0.8600
S2—O5	1.485 (3)	N4—H43	0.8600
S2—O6	1.503 (2)	C1—C2	1.514 (5)
S3—O11	1.437 (3)	C1—H1A	0.9700
S3—O12	1.441 (3)	C1—H1B	0.9700
S3—O10	1.508 (3)	C2—C3	1.510 (5)
S3—O9	1.524 (3)	C2—H2A	0.9700
S4—O15	1.448 (3)	C2—H2B	0.9700
S4—O16	1.449 (3)	C3—H3A	0.9700
S4—O14	1.492 (2)	C3—H3B	0.9700
S4—O13	1.505 (2)	C4—C5	1.496 (6)
O8—Ce1^ii^	2.352 (2)	C4—H4A	0.9700
O1w—H1w1	0.8368	C4—H4B	0.9700
O1w—H1w2	0.8351	C5—C6	1.486 (6)
O2w—H2w1	0.8401	C5—H5A	0.9700
O2w—H2w2	0.8390	C5—H5B	0.9700
O3w—H3w1	0.8444	C6—H6A	0.9700
O3w—H3w2	0.8478	C6—H6B	0.9700
			
O1—Ce1—O8^i^	79.66 (9)	S4—O14—Ce1	99.66 (12)
O1—Ce1—O9	91.34 (9)	Ce1—O1w—H1w1	110.1
O8^i^—Ce1—O9	126.76 (9)	Ce1—O1w—H1w2	132.2
O1—Ce1—O1w	77.33 (9)	H1w1—O1w—H1w2	109.9
O8^i^—Ce1—O1w	83.33 (9)	H2w1—O2w—H2w2	109.4
O9—Ce1—O1w	145.79 (8)	H3w1—O3w—H3w2	107.6
O1—Ce1—O13	148.37 (9)	C1—N1—H11	109.5
O8^i^—Ce1—O13	81.78 (9)	C1—N1—H12	109.5
O9—Ce1—O13	79.59 (8)	H11—N1—H12	109.5
O1w—Ce1—O13	125.54 (8)	C1—N1—H13	109.5
O1—Ce1—O14	138.98 (8)	H11—N1—H13	109.5
O8^i^—Ce1—O14	75.94 (9)	H12—N1—H13	109.5
O9—Ce1—O14	129.67 (8)	C3—N2—H21	109.5
O1w—Ce1—O14	67.56 (8)	C3—N2—H22	109.5
O13—Ce1—O14	58.03 (8)	H21—N2—H22	109.5
O1—Ce1—O10	76.20 (9)	C3—N2—H23	109.5
O8^i^—Ce1—O10	68.77 (8)	H21—N2—H23	109.5
O9—Ce1—O10	58.17 (8)	H22—N2—H23	109.5
O1w—Ce1—O10	144.34 (9)	C4—N3—H31	109.5
O13—Ce1—O10	73.27 (8)	C4—N3—H32	109.5
O14—Ce1—O10	122.81 (8)	H31—N3—H32	109.5
O1—Ce1—O6	129.18 (9)	C4—N3—H33	109.5
O8^i^—Ce1—O6	146.41 (8)	H31—N3—H33	109.5
O9—Ce1—O6	75.37 (8)	H32—N3—H33	109.5
O1w—Ce1—O6	86.94 (8)	C6—N4—H41	109.5
O13—Ce1—O6	78.00 (8)	C6—N4—H42	109.5
O14—Ce1—O6	70.63 (8)	H41—N4—H42	109.5
O10—Ce1—O6	128.51 (8)	C6—N4—H43	109.5
O1—Ce1—O5	72.31 (9)	H41—N4—H43	109.5
O8^i^—Ce1—O5	145.65 (8)	H42—N4—H43	109.5
O9—Ce1—O5	74.19 (8)	N1—C1—C2	109.4 (3)
O1w—Ce1—O5	71.61 (9)	N1—C1—H1A	109.8
O13—Ce1—O5	131.94 (8)	C2—C1—H1A	109.8
O14—Ce1—O5	113.52 (9)	N1—C1—H1B	109.8
O10—Ce1—O5	121.19 (9)	C2—C1—H1B	109.8
O6—Ce1—O5	56.88 (8)	H1A—C1—H1B	108.2
O3—S1—O4	111.3 (2)	C3—C2—C1	113.3 (3)
O3—S1—O2	110.1 (2)	C3—C2—H2A	108.9
O4—S1—O2	111.7 (2)	C1—C2—H2A	108.9
O3—S1—O1	109.16 (17)	C3—C2—H2B	108.9
O4—S1—O1	106.74 (18)	C1—C2—H2B	108.9
O2—S1—O1	107.59 (16)	H2A—C2—H2B	107.7
O7—S2—O8	112.34 (16)	N2—C3—C2	113.0 (3)
O7—S2—O5	113.34 (18)	N2—C3—H3A	109.0
O8—S2—O5	108.60 (15)	C2—C3—H3A	109.0
O7—S2—O6	111.47 (17)	N2—C3—H3B	109.0
O8—S2—O6	107.82 (14)	C2—C3—H3B	109.0
O5—S2—O6	102.71 (14)	H3A—C3—H3B	107.8
O11—S3—O12	113.18 (17)	N3—C4—C5	111.7 (4)
O11—S3—O10	111.37 (17)	N3—C4—H4A	109.3
O12—S3—O10	111.43 (16)	C5—C4—H4A	109.3
O11—S3—O9	109.67 (17)	N3—C4—H4B	109.3
O12—S3—O9	110.01 (16)	C5—C4—H4B	109.3
O10—S3—O9	100.44 (14)	H4A—C4—H4B	107.9
O15—S4—O16	111.8 (2)	C6—C5—C4	111.5 (4)
O15—S4—O14	110.90 (17)	C6—C5—H5A	109.3
O16—S4—O14	110.85 (16)	C4—C5—H5A	109.3
O15—S4—O13	110.46 (17)	C6—C5—H5B	109.3
O16—S4—O13	110.17 (17)	C4—C5—H5B	109.3
O14—S4—O13	102.30 (14)	H5A—C5—H5B	108.0
S1—O1—Ce1	145.18 (15)	N4—C6—C5	113.2 (4)
S2—O5—Ce1	99.61 (12)	N4—C6—H6A	108.9
S2—O6—Ce1	100.49 (12)	C5—C6—H6A	108.9
S2—O8—Ce1^ii^	144.13 (15)	N4—C6—H6B	108.9
S3—O9—Ce1	101.91 (12)	C5—C6—H6B	108.9
S3—O10—Ce1	99.48 (12)	H6A—C6—H6B	107.7
S4—O13—Ce1	99.86 (12)		
			
O3—S1—O1—Ce1	96.8 (3)	O13—Ce1—O9—S3	76.58 (13)
O4—S1—O1—Ce1	−142.7 (3)	O14—Ce1—O9—S3	108.19 (13)
O2—S1—O1—Ce1	−22.7 (4)	O10—Ce1—O9—S3	−0.09 (11)
O8^i^—Ce1—O1—S1	112.7 (3)	O6—Ce1—O9—S3	156.76 (14)
O9—Ce1—O1—S1	−120.1 (3)	O5—Ce1—O9—S3	−144.11 (14)
O1w—Ce1—O1—S1	27.3 (3)	O11—S3—O10—Ce1	−116.21 (16)
O13—Ce1—O1—S1	167.8 (2)	O12—S3—O10—Ce1	116.37 (15)
O14—Ce1—O1—S1	58.6 (3)	O9—S3—O10—Ce1	−0.13 (15)
O10—Ce1—O1—S1	−176.8 (3)	O1—Ce1—O10—S3	100.51 (14)
O6—Ce1—O1—S1	−48.0 (3)	O8^i^—Ce1—O10—S3	−175.39 (15)
O5—Ce1—O1—S1	−47.2 (3)	O9—Ce1—O10—S3	0.09 (11)
O7—S2—O5—Ce1	−115.29 (16)	O1w—Ce1—O10—S3	143.65 (13)
O8—S2—O5—Ce1	119.11 (13)	O13—Ce1—O10—S3	−87.88 (13)
O6—S2—O5—Ce1	5.10 (15)	O14—Ce1—O10—S3	−119.49 (13)
O1—Ce1—O5—S2	177.08 (15)	O6—Ce1—O10—S3	−28.99 (18)
O8^i^—Ce1—O5—S2	140.24 (13)	O5—Ce1—O10—S3	41.46 (16)
O9—Ce1—O5—S2	−86.27 (13)	O15—S4—O13—Ce1	−114.54 (17)
O1w—Ce1—O5—S2	94.93 (14)	O16—S4—O13—Ce1	121.47 (16)
O13—Ce1—O5—S2	−26.73 (18)	O14—S4—O13—Ce1	3.56 (15)
O14—Ce1—O5—S2	40.62 (15)	O1—Ce1—O13—S4	−135.59 (15)
O10—Ce1—O5—S2	−121.98 (13)	O8^i^—Ce1—O13—S4	−81.04 (13)
O6—Ce1—O5—S2	−3.67 (11)	O9—Ce1—O13—S4	149.06 (14)
O7—S2—O6—Ce1	116.49 (16)	O1w—Ce1—O13—S4	−5.37 (17)
O8—S2—O6—Ce1	−119.76 (13)	O14—Ce1—O13—S4	−2.54 (11)
O5—S2—O6—Ce1	−5.19 (15)	O10—Ce1—O13—S4	−151.25 (14)
O1—Ce1—O6—S2	4.56 (17)	O6—Ce1—O13—S4	71.98 (13)
O8^i^—Ce1—O6—S2	−139.44 (13)	O5—Ce1—O13—S4	91.57 (15)
O9—Ce1—O6—S2	84.09 (12)	O15—S4—O14—Ce1	114.25 (18)
O1w—Ce1—O6—S2	−66.35 (12)	O16—S4—O14—Ce1	−120.97 (16)
O13—Ce1—O6—S2	166.31 (13)	O13—S4—O14—Ce1	−3.54 (15)
O14—Ce1—O6—S2	−133.63 (14)	O1—Ce1—O14—S4	146.82 (12)
O10—Ce1—O6—S2	109.35 (13)	O8^i^—Ce1—O14—S4	91.56 (13)
O5—Ce1—O6—S2	3.63 (11)	O9—Ce1—O14—S4	−34.86 (17)
O7—S2—O8—Ce1^ii^	9.8 (3)	O1w—Ce1—O14—S4	−179.93 (15)
O5—S2—O8—Ce1^ii^	136.0 (2)	O13—Ce1—O14—S4	2.56 (11)
O6—S2—O8—Ce1^ii^	−113.4 (3)	O10—Ce1—O14—S4	38.84 (16)
O11—S3—O9—Ce1	117.48 (15)	O6—Ce1—O14—S4	−85.13 (13)
O12—S3—O9—Ce1	−117.42 (15)	O5—Ce1—O14—S4	−123.43 (12)
O10—S3—O9—Ce1	0.13 (15)	N1—C1—C2—C3	168.6 (3)
O1—Ce1—O9—S3	−72.92 (13)	C1—C2—C3—N2	70.0 (5)
O8^i^—Ce1—O9—S3	5.16 (17)	N3—C4—C5—C6	172.7 (4)
O1w—Ce1—O9—S3	−142.07 (13)	C4—C5—C6—N4	−174.8 (4)

Symmetry codes: (i) *x*, −*y*+1/2, *z*+1/2; (ii) *x*, −*y*+1/2, *z*−1/2.

### Hydrogen-bond geometry (Å, °)

**Table:**

*D*—H···*A*	*D*—H	H···*A*	*D*···*A*	*D*—H···*A*
O1w—H1w2···O12^iii^	0.84	2.05	2.854 (4)	162
O2w—H2w1···O11^iii^	0.84	2.24	2.935 (5)	140
O2w—H2w2···O15^iv^	0.84	2.08	2.902 (5)	167
O3w—H3w1···O3^iii^	0.84	1.90	2.729 (5)	169
O3w—H3w2···O2w	0.85	1.98	2.802 (5)	163
N1—H11···O9^iii^	0.86	2.04	2.881 (4)	167
N1—H12···O3w	0.86	1.95	2.806 (5)	171
N1—H13···O13^v^	0.86	2.19	3.036 (4)	166
N2—H21···O16^ii^	0.86	2.09	2.904 (5)	157
N2—H22···O16^iv^	0.86	2.25	2.978 (5)	142
N2—H23···O2	0.86	2.49	3.180 (6)	137
N2—H23···O5	0.86	2.44	2.989 (4)	122
N3—H31···O6^i^	0.86	2.04	2.866 (4)	162
N3—H32···O4	0.86	2.37	2.882 (6)	119
N3—H32···O2w^vi^	0.86	2.37	3.085 (6)	141
N3—H33···O1	0.86	2.33	3.004 (5)	135
N3—H33···O10	0.86	2.43	3.191 (6)	147
N4—H41···O6^vii^	0.86	2.34	2.986 (4)	132
N4—H41···O15^vii^	0.86	2.25	2.951 (5)	138
N4—H43···O4^viii^	0.86	2.00	2.851 (5)	173

Symmetry codes: (iii) *x*−1, *y*, *z*; (iv) −*x*+1, *y*−1/2, −*z*+1/2; (v) *x*−1, −*y*+1/2, *z*−1/2; (ii) *x*, −*y*+1/2, *z*−1/2; (i) *x*, −*y*+1/2, *z*+1/2; (vi) −*x*+1, −*y*, −*z*+1; (vii) *x*+1, −*y*+1/2, *z*+1/2; (viii) −*x*+2, −*y*, −*z*+1.
